# Hounsfield unit for assessing asymmetrical loss of vertebral bone mineral density and its correlation with curve severity in adolescent idiopathic scoliosis

**DOI:** 10.3389/fsurg.2022.1000031

**Published:** 2022-09-22

**Authors:** Yunzhong Cheng, Honghao Yang, Yong Hai, Aixing Pan, Yaoshen Zhang, Lijin Zhou

**Affiliations:** Department of Orthopedic Surgery, Beijing Chao-Yang Hospital, Capital Medical University, Beijing, China

**Keywords:** vertebral bone mineral density, Hounsfield unit, adolescent idiopathic scoliosis, low bone mass, computed tomography

## Abstract

**Background:**

Low bone mass concomitantly occurs in patients with adolescent idiopathic scoliosis (AIS) and can persist until skeletal maturity. The purpose of this study was to assess the asymmetrical loss of vertebral bone mineral density (vBMD) and its correlation with curve severity in patients with AIS using Hounsfield unit (HU) values measured from computed tomography scans.

**Methods:**

A total of 93 AIS patients were retrospectively recruited. The HU values of the vertebral body (VB-HU) and pedicle screw trajectory (PST-HU) were measured from four vertebrae above (Apex − 4) to four below (Apex + 4) the apical vertebra (Apex) of the major curve. The VB-HU and PST-HU at the upper end vertebra, Apex, and lower end vertebra within the concave and convex sides of the major and minor curves and stable vertebrae were obtained.

**Results:**

A significant correlation was found between the Cobb angle and VB-HU at the periapical levels of the major curve. VB-HU and PST-HU at periapical levels were significantly greater within the concavity than the convexity of both major and minor curves. The asymmetric ratios of VB-HU and PST-HU were significantly correlated with the major curve Cobb angle, peaked at the apex, and gradually diminished from the apex to the end vertebrae. The asymmetrical loss of vBMD aggravated with the progression of curve severity, presenting as VB-HU, significantly decreased within the convexity and insignificantly decreased within the concavity of the major curve.

**Conclusion:**

The asymmetrical loss of vBMD was associated with the progression of curve severity in AIS. For patients with severe AIS, the distraction of the pedicle screws at the concave side should be a priority in correcting the major curve, and supplemental anchors and larger-sized screws should be placed within the convex side around the apex of the major curve to reduce the risk of screw loosening after surgery.

## Introduction

Adolescent idiopathic scoliosis (AIS) is a three-dimensional spinal deformity affecting children aged 10 years to maturity (1). AIS can manifest as a disturbed self-image, deformity progression, early back degeneration, and cardiopulmonary compromises (2). Therapeutic options include a brace and specific exercises for mild scoliosis and surgical instrumentation and spinal fusion for severe or rapidly progressive curves (3). Low bone mass concomitantly occurred with the spinal deformity and could still exist after skeletal maturity (4). The prevalence of low bone mass in AIS was 25%–31% and was the culprit for curve progression and implant loosening (2, 5–7).

A dual-energy x-ray absorptiometry (DXA) scan is the gold standard for measuring bone mineral water density (BMD) and diagnosing osteopenia or osteoporosis in clinical practice (8). However, DXA could not discover BMD fluctuations between cancellous and cortical bone, which would impact the accuracy of bone quality evaluation (9). Meanwhile, vertebral rotation by scoliosis could make the outcome of DXA-based BMD evaluation unreliable (10). Although quantitative computed tomography (QCT) can accurately focus on the cancellous bone area and accommodate vertebral rotation by setting slicing planes, it is impractical to apply this technique in many clinical settings due to the high-cost equipment and need for rigorous training (11).

Hounsfield unit (HU) measured from CT scans has been widely reported as a valuable technique for evaluating vertebral bone mineral density (vBMD) in recent years, and it is closely correlated with standard BMD and compressive strength (12). A CT scan is a routine preoperative examination for AIS, and practitioners can easily obtain additional information on areal bone mineral density at no extra cost. However, no previous studies have investigated vBMD using HU measurements for patients with AIS.

The purpose of this study was to assess the asymmetrical loss of vBMD and its correlation with curve severity in AIS patients.

## Methods

### Subjects

A retrospective consecutive case review was performed to identify patients with AIS between January 2014 and December 2020 in our hospital. The inclusion criteria were as follows: (i) age from 10 to 20 years; (ii) the main thoracic curve (MTC) was the major curve (Lenke 1–4); (iii) full-spine posterior–anterior radiography; and (iv) full-spine CT scan for HU measurement. The exclusion criteria were as follows: (i) history of spinal surgery; (ii) spinal infections or metabolic disease; and (iii) other pathogenesis of scoliosis (e.g., congenital). A total of 93 patients met both the inclusion and exclusion criteria.

### Data collection

The demographic data, including age, sex, and body mass index (BMI), were recorded. The Cobb angle of the major curve and the two minor curves, the proximal thoracic curve (PTC) and thoracolumbar/lumbar curve (TLC), were evaluated based on full-spine posterior–anterior radiography. The BMD of the L4 vertebra was examined by a DXA scan, and a T-score ≤ −2.0 was used to distinguish low bone mass from normal bone density (13).

### HU measurement

Full-spine CT scans were performed in the supine position with the following parameters: 320 mAs, 120 kVP, and 5 mm thickness. All Digital Imaging and Communications in Medicine (DICOM) data were analyzed using Horos software (Horos; v3.3.1@horosproject).

**Figure 1:**
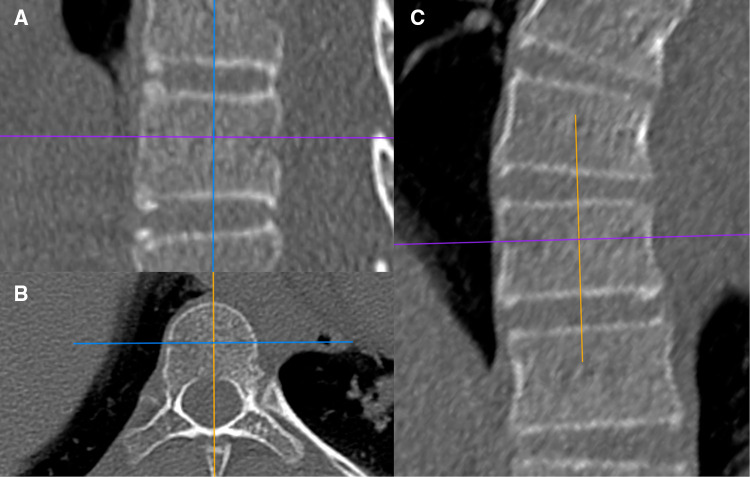
Schematic diagram of the sagittal slicing plane (**A**), the transverse slicing plane (**B**), and the coronal slicing plane (**C**).

**Figure 2:**
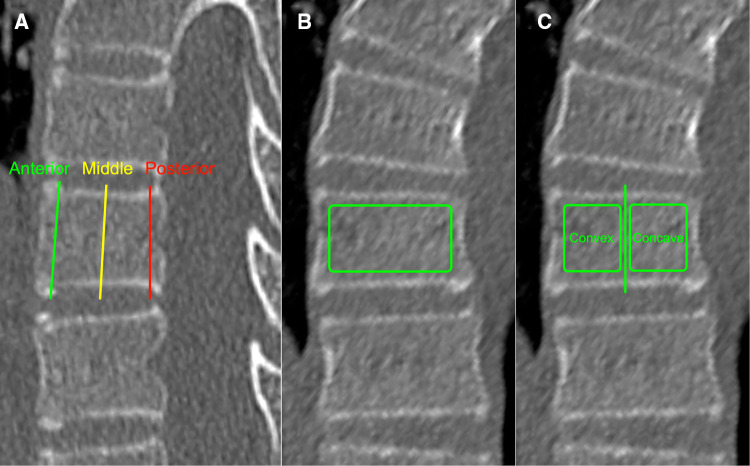
Schematic diagram of VB-HU measurement; three measurement locations were set on the sagittal plane (**A**): immediately posterior to the anterior vertebral cortex, in the middle of the vertebral body, and immediately anterior to the posterior vertebral cortex. The VB-HU of the whole vertebra (**B**) and the VB-HU within concave and convex sides (**C**) were obtained on the coronal plane.

The HU values of each vertebral body (VB-HU) were measured using the method described by Wang et al. (14), with some modifications. The slicing plane was parallel to the superior vertebral end plate on coronal and sagittal planes and through the midpoint of the posterior edge of the spinal canal and the anterior edge of the vertebral body on the transverse plane (Figure 1). The region of interest (ROI) was placed on the coronal images of the vertebral body. The VB-HU was obtained at three locations on the coronal plane: immediately posterior to the anterior vertebral cortex, in the middle of the vertebral body, and immediately anterior to the posterior vertebral cortex. The ROI was rectangular, as large as possible, excluding the cortical margins to prevent volume averaging (12). Then, the VB-HU within the concave and convex sides were obtained separately (Figure 2). The VB-HU measured from the three slicing planes was averaged to calculate the mean HU for each vertebral body.

**Figure 3:**
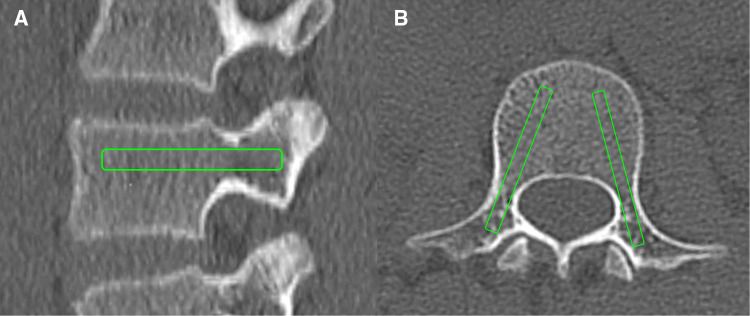
Schematic diagram of PST-HU measurement: the PST-HU was obtained on the sagittal plane (**A**) and the transverse plane (**B**).

The HU values of each pedicle screw trajectory (PST-HU) were measured using the methods described by Ishikawa et al. and Zhang et al. (15, 16). The slicing plane was adjusted along the PST on the transverse plane. The rectangular ROI was placed on the sagittal and transverse images of the pedicle screw trajectory inside the cortical shell (Figure 3). The size of the ROI was selected as follows: 25 or 30 mm  ×  4.0 or 4.5 mm from T1 to T5; 30 or 35 mm  ×  4.0 or 4.5 mm from T6 to T9; 30 or 35 mm × 4.5 or 5.0 mm from T10 to T12; 35 or 40 mm × 5.0 or 5.5 mm from L1 to L5 (17). The HU values measured from the sagittal and transverse planes were averaged to calculate the mean PST-HU for each pedicle screw trajectory.

VB-HU and PST-HU were measured from four vertebrae above (Apex − 4) to four below (Apex + 4) the apical vertebra (Apex) of the major curve. The data at the upper end vertebra (UEV), upper stable vertebra (USV), lower end vertebra (LEV), and lower stable vertebra (LSV) were also obtained. For the minor curves, the VB-HU and PST-HU at the apex, UEV, and LEV were measured. All parameters were measured by two independent observers and averaged for statistical analysis. The asymmetric ratios of VB-HU and PST-HU were calculated by HU values within the concave side/HU values within the convex side.

### Statistical analysis

All statistical analyses were performed utilizing SPSS version 25.0 (Chicago, IL, USA). Continuous variables were presented as the mean ± standard deviation. The difference in HU values between the concave and convex sides within the target vertebral level was analyzed using a paired *t*-test. The asymmetric HU ratio among subgroups was compared using one-way ANOVA. The Pearson correlation coefficient was calculated to evaluate the correlation between the Cobb angle of the major curve and HU-VB as well as the asymmetric HU ratio. Differences were considered statistically significant when *P* < 0.05.

## Results

### Characteristics of the subjects

Among the 93 patients recruited in the current study, 35 were males and 58 were females, with a mean age of 14.6 ± 2.4 years. The mean BMI was 21.9 ± 2.3 kg/m^2^. The mean Cobb angle of MTC was 75.9° ± 29.6°, with PTC of 37.2° ± 14.5° and TLC of 41.5° ± 16.2°. The distributions of UEV, Apex, and LEV of the major and minor curves are shown in Table 1. A total of 43.0% (40/93) of patients presented low bone mass at L4 (T-score ≤ −2.0), and 57.0% (53/93) presented normal bone density.

**Table 1:** Distribution of UEV, Apex, and LEV of major and minor curves.

	PTC			MTC			TLC		
	UEV	Apex	LEV	UEV	Apex	LEV	UEV	Apex	LEV
T1	66 (71.0%)								
T2	26 (28.0%)	56 (60.2%)							
T3	1 (1.1%)	32 (34.4%)	2 (2.2%)	2 (2.2%)					
T4		5 (5.4%)	27 (29.0%)	8 (8.6%)					
T5			32 (34.4%)	27 (29.0%)					
T6			25 (26.9%)	31 (33.3%)	2 (2.2%)				
T7			6 (6.5%)	23 (24.7%)	7 (7.5%)				
T8			1 (1.1%)	2 (2.2%)	24 (25.8%)	2 (2.2%)			
T9					39 (41.9%)	2 (2.2%)	2 (2.2%)		
T10					20 (21.5%)	5 (5.4%)	3 (3.2%)		
T11					1 (1.1%)	29 (31.2%)	14 (15.1%)		
T12						31 (33.3%)	42 (45.2%)	1 (1.1%)	
L1						19 (20.4%)	23 (24.7%)	9 (9.7%)	
L2						5 (5.4%)	9 (9.7%)	18 (19.4%)	3 (3.2%)
L3								49 (52.7%)	11 (11.8%)
L4								16 (17.2%)	45 (48.4%)
L5									34 (36.6%)

PTC, proximal thoracic curve; MTC, main thoracic curve; TLC, thoracolumbar/lumbar curve; UEV, upper end vertebra; Apex, apical vertebra; LEV, lower end vertebra.

### Mean HU values and asymmetric ratio from Apex − 4 to Apex + 4 of the major curve

The mean VB-HU from Apex − 4 to Apex + 4 of the major curve is shown in Table 2. No significant difference among vertebral levels was detected. There were significantly negative moderate correlations between the mean VB-HU at each level and the Cobb angle.

**Table 2:** VB-HU from Apex − 4 to Apex + 4 of the major curve and its correlation with Cobb angle.

Level	VB-HU	Correlation with Cobb angle	
		Pearson correlation coefficient	*P*
Apex − 4	244.1 ± 40.0	−0.533	<0.01
Apex − 3	237.9 ± 40.7	−0.534	<0.01
Apex − 2	240.0 ± 40.6	−0.588	<0.01
Apex − 1	244.2 ± 41.1	−0.635	<0.01
Apex	242.8 ± 41.9	−0.709	<0.01
Apex + 1	244.5 ± 43.5	−0.600	<0.01
Apex + 2	244.2 ± 45.0	−0.563	<0.01
Apex + 3	245.4 ± 44.4	−0.532	<0.01
Apex + 4	246.3 ± 46.2	−0.512	<0.01

VB-HU, the Hounsfield Unit values of vertebral body; Apex, apical vertebra.

VB-HU and PST-HU within the concave and convex sides and their asymmetric ratios are shown in Table 3. The VB-HU and PST-HU within the concave side were significantly greater than those within the convex side from Apex − 3 to Apex + 3. However, at Apex − 4 and Apex + 4, the VB-HU and PST-HU within the convex side were statistically greater than those within the concave side, which was contrary to other vertebral levels. The asymmetric ratio of VB-HU and PST-HU peaked at the apex, with values of 1.43 ± 0.27 and 1.40 ± 0.25, respectively. The asymmetric ratio indicated a declining tendency cranially and caudally and decreased gradually to a minimum at Apex − 4 and Apex + 4.

**Table 3:** VB-HU and PST-HU within the concave and convex side of the major curve and its asymmetric ratio.

	VB-HU				PST-HU			
Level	Concave	Convex	Ratio	*P*	Concave	Convex	Ratio	*P*
Apex − 4	233.9 ± 39.6	255.2 ± 44.2	0.92 ± 0.08	<0.001	239.1 ± 38.5	269.6 ± 43.1	0.89 ± 0.09	<0.001
Apex − 3	241.8 ± 41.0	233.3 ± 44.9	1.05 ± 0.12	0.001	249.6 ± 36.2	250.9 ± 45.5	1.01 ± 0.14	0.607
Apex − 2	256.4 ± 38.8	221.0 ± 45.6	1.18 ± 0.14	<0.001	266.0 ± 39.5	236.2 ± 45.6	1.15 ± 0.14	<0.001
Apex − 1	271.3 ± 39.4	213.4 ± 48.2	1.31 ± 0.22	<0.001	281.3 ± 40.7	225.3 ± 45.9	1.28 ± 0.18	<0.001
Apex	280.8 ± 39.4	203.7 ± 48.4	1.43 ± 0.27	<0.001	289.6 ± 39.7	213.3 ± 47.3	1.40 ± 0.25	<0.001
Apex + 1	269.6 ± 42.8	214.8 ± 50.2	1.29 ± 0.20	<0.001	278.2 ± 44.1	219.6 ± 47.8	1.30 ± 0.21	<0.001
Apex + 2	259.3 ± 45.5	224.6 ± 49.5	1.18 ± 0.15	<0.001	270.2 ± 42.8	231.5 ± 45.8	1.19 ± 0.16	<0.001
Apex + 3	245.0 ± 44.7	242.2 ± 48.4	1.02 ± 0.11	0.164	253.3 ± 40.3	243.3 ± 44.2	1.05 ± 0.14	<0.001
Apex + 4	235.9 ± 48.1	251.0 ± 48.3	0.94 ± 0.09	<0.001	237.8 ± 41.2	251.2 ± 43.4	0.95 ± 0.10	<0.001

VB-HU, the Hounsfield Unit values of vertebral body; PST-HU, the Hounsfield Unit values of pedicle screw trajectory; Apex, apical vertebra.

### HU values and asymmetric ratio at the UEV, Apex, LEV, USV, and LSV

HU values and asymmetric HU ratios at UEV, Apex, LEV, USV, and LSV are shown in Table 4. The VB-HU within the concave side was significantly greater than that on the convex side at the apex, UEV, and LEV of MTC. The PST-HU was also significantly greater within the concave side at the apex and LEV. However, there was no significant difference in HU values between the concave and convex sides at USV and LSV.

**Table 4:** HU values and the asymmetric ratio at UEV, Apex, and LEV of major and minor curves.

	VB-HU				PST-HU			
Level	Concave	Convex	Ratio	*P*	Concave	Convex	Ratio	*P*
PTC								
UEV	254.6 ± 44.1	254.1 ± 44.1	1.00 ± 0.03	0.539	268.1 ± 44.1	267.5 ± 43.4	1.00 ± 0.03	0.444
Apex	278.3 ± 45.4	248.7 ± 48.4	1.13 ± 0.11	<0.001	287.0 ± 43.1	261.6 ± 45.6	1.11 ± 0.08	<0.001
LEV	250.0 ± 43.5	236.6 ± 39.9	1.06 ± 0.10	<0.001	266.5 ± 42.4	244.1 ± 38.2	1.10 ± 0.12	<0.001
USV	243.7 ± 40.2	240.3 ± 43.5	1.01 ± 0.09	0.773	249.6 ± 41.5	255.0 ± 41.7	0.98 ± 0.08	0.251
MTC								
UEV	243.2 ± 42.2	236.1 ± 42.2	1.04 ± 0.10	0.003	252.2 ± 37.0	253.5 ± 41.0	1.00 ± 0.11	0.616
Apex	280.8 ± 39.4	203.7 ± 48.4	1.43 ± 0.27	<0.001	289.6 ± 39.7	213.3 ± 47.3	1.40 ± 0.25	<0.001
LEV	246.1 ± 49.2	240.4 ± 47.7	1.03 ± 0.10	0.01	255.1 ± 45.2	244.4 ± 42.7	1.05 ± 0.12	<0.001
LSV	233.8 ± 46.3	239.2 ± 45.9	0.98 ± 0.07	0.089	237.8 ± 43.7	240.9 ± 43.0	0.99 ± 0.07	0.263
TLC								
UEV	244.8 ± 48.2	240.4 ± 47.3	1.02 ± 0.09	0.250	247.2 ± 41.4	246.2 ± 42.2	1.01 ± 0.09	0.652
Apex	252.2 ± 51.5	224.4 ± 49.3	1.13 ± 0.10	<0.001	263.2 ± 48.3	235.9 ± 47.2	1.12 ± 0.10	<0.001
LEV	244.5 ± 45.5	243.5 ± 44.9	1.00 ± 0.04	0.253	254.4 ± 43.2	254.1 ± 43.7	1.00 ± 0.03	0.704

VB-HU, the Hounsfield Unit values of vertebral body; PST-HU, the Hounsfield Unit values of pedicle screw trajectory; PTC, proximal thoracic curve; MTC, main thoracic curve; TLC, thoracolumbar/lumbar curve; UEV, upper end vertebra; Apex, apical vertebra; LEV, lower end vertebra; USV, upper stable vertebra; LSV, lower stable vertebra.

For PTC, VB-HU and PST-HU within the concave side were significantly greater than those within the convex side at the apex and LEV, while no significant difference was detected at the UEV. For TLC, the VB-HU and PST-HU were significantly greater within the concave side at the apex, while there was no difference at UEV and LEV.

**Figure 4:**
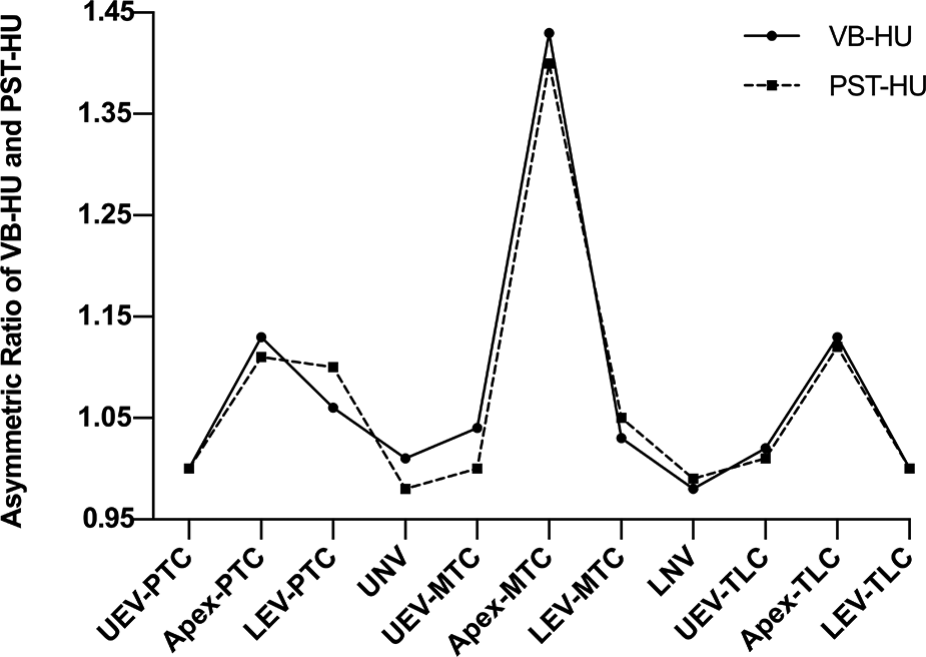
Asymmetric ratio of VB-HU and PST-HU at UEV, Apex, LEV, USV, and LSV.

The asymmetric HU ratio peaked at the apex of both major and minor curves and gradually diminished from the apex to the end vertebrae (Figure 4). The asymmetric HU ratio at the apex of the major curve was significantly greater than that of the minor curves (*P* < 0.001). There was no significant difference in the asymmetric HU ratio at UEV and LEV among all curves.

### Comparison of VB-HU at the Apex, UEV, and LEV of the major curve among subgroups

**Figure 5:**
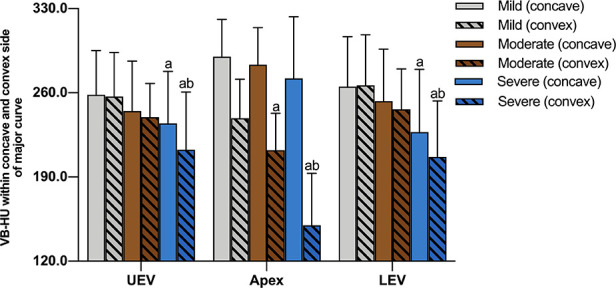
Comparison of VB-HU at Apex, UEV, and LEV of the major curve among subgroups; a indicates a significant difference compared with the mild group; b indicates a significant difference compared with the moderate group.

The 93 patients were divided into three groups according to the Cobb angle of the major curve: mild group (<60°), moderate group (60°–90°), and severe group (>90°). The comparison of VB-HU at Apex, UEV, and LEV of the major curve among subgroups is shown in Table 5. There was a significant difference in VB-HU at the apex among the subgroups. Although the VB-HU within the concave side at the apex gradually decreased with increasing Cobb angle, there was no significant difference among the subgroups. However, the mean VB-HU and the VB-HU within the convex side at the apex significantly decreased from the mild to severe group (Figure 5). No significant difference was detected in VB-HU at UEV and LEV between the mild and moderate groups, while VB-HU in the severe group was significantly less than that in the other groups. Between the mild and moderate groups, the VB-HU within neither side was significantly different at UEV and LEV. In the severe group, the VB-HU within the concave side was significantly less than that in the mild group at UEV and LEV, and the VB-HU within the convex side was significantly less than that in the other groups.

**Table 5:** Comparison of VB-HU at Apex, UEV, and LEV within major curve among subgroups.

	<60° (*n* = 31)			60°–90° (*n* = 31)			>90° (*n* = 31)		
Level	Mean	Concave	Convex	Mean	Concave	Convex	Mean	Concave	Convex
UEV	258.6 ± 35.5	258.3 ± 36.7	256.9 ± 36.6	241.3 ± 30.2	244.8 ± 41.6	239.8 ± 27.9	218.8 ± 42.0^ab^	234.5 ± 43.2^a^	212.6 ± 48.0^ab^
Apex	268.5 ± 29.4	290.0 ± 31.0	238.9 ± 32.4	241.2 ± 27.0^a^	283.4 ± 30.8	212.3 ± 30.7^a^	208.8 ± 42.9^ab^	272.0 ± 51.2	149.9 ± 43.2^ab^
LEV	266.2 ± 40.0	265.2 ± 41.4	266.2 ± 42.0	253.3 ± 38.7	253.0 ± 43.3	246.2 ± 33.7	216.7 ± 44.6^ab^	227.4 ± 52.1^a^	206.7 ± 46.5^ab^

^a^ indicates a significant difference compared with <60° group.

^b^ indicates a significant difference compared with 60°–90° group.

VB-HU, the Hounsfield Unit values of vertebral body; UEV, upper end vertebra; Apex, apical vertebra; LEV, lower end vertebra.

### Correlation between the asymmetric HU ratio and Cobb angle of the major curve

The correlation between the asymmetric HU ratio and the Cobb angle of the major curve is shown in Table 6. There were significantly positive and strong correlations between the Cobb angle and the asymmetric ratio of VB-HU and PST-HU at the apex of the major curve (*r* = 0.880, *P* < 0.001; *r* = 0.758, *P* < 0.001). The asymmetric ratios of VB-HU and PST-HU at the apex of the minor curves were also significantly associated with the Cobb angle of the major curve.

**Table 6:** Correlation between asymmetric HU ratio and cobb angle of major and minor curves.

	Asymmetric ratio of VB-HU			Correlation		Asymmetric ratio of PST-HU			Correlation	
Level	<60° (*n* = 31)	60–90° (*n* = 31)	>90° (*n* = 31)	*r*	*P*	<60° (*n* = 31)	60–90° (*n* = 31)	>90° (*n* = 31)	*r*	*P*
PTC										
UEV	1.00 ± 0.03	1.00 ± 0.03	1.01 ± 0.04	0.089	0.399	1.00 ± 0.02	1.00 ± 0.03	1.01 ± 0.04	0.028	0.791
Apex	1.08 ± 0.08	1.13 ± 0.07	1.20 ± 0.14^ab^	0.463	<0.001	1.06 ± 0.07	1.10 ± 0.05	1.17 ± 0.10^ab^	0.477	<0.001
LEV	1.00 ± 0.13	1.03 ± 0.09	1.06 ± 0.09	0.258	0.053	1.02 ± 0.08	1.10 ± 0.12	1.08 ± 0.12	0.200	0.054
MTC										
UEV	1.01 ± 0.07	1.02 ± 0.10	1.10 ± 0.11^ab^	0.319	0.002	0.97 ± 0.07	0.98 ± 0.09	1.08 ± 0.15^ab^	0.351	0.001
Apex	1.22 ± 0.08	1.35 ± 0.14^a^	1.83 ± 0.22^ab^	0.880	<0.001	1.23 ± 0.08	1.36 ± 0.15^a^	1.79 ± 0.28^ab^	0.758	<0.001
LEV	1.00 ± 0.07	1.03 ± 0.09	1.09 ± 0.12^a^	0.306	0.003	1.01 ± 0.06	1.05 ± 0.11	1.09 ± 0.18	0.231	0.026
TLC										
UEV	1.01 ± 0.05	1.02 ± 0.08	1.01 ± 0.12	−0.147	0.159	1.01 ± 0.06	1.01 ± 0.09	1.00 ± 0.14	−0.101	0.336
Apex	1.06 ± 0.07	1.15 ± 0.08^a^	1.20 ± 0.12^a^	0.469	<0.001	1.06 ± 0.06	1.14 ± 0.08^a^	1.18 ± 0.11^a^	0.404	<0.001
LEV	1.00 ± 0.03	1.00 ± 0.03	1.01 ± 0.05	0.072	0.494	1.00 ± 0.01	1.00 ± 0.03	1.01 ± 0.04	0.101	0.336

^a^ indicates a significant difference compared with the <60° group.

^b^ indicates a significant difference compared with the 60°–90° group.

VB-HU, the Hounsfield Unit values of vertebral body; PST-HU, the Hounsfield Unit values of pedicle screw trajectory; PTC, proximal thoracic curve; MTC, main thoracic curve; TLC, thoracolumbar/lumbar curve; UEV, upper end vertebra; Apex, apical vertebra; LEV, lower end vertebra.

### Comparison of asymmetric HU ratios among subgroups

A comparison of the asymmetric HU ratio among subgroups is shown in Table 6. There was a significant difference in the asymmetric ratio of VB-HU and PST-HU at the apex of the major curve among the subgroups. The ratio at UEV was significantly greater in the severe group, while there was no significant difference between the mild and moderate groups. The only difference in the asymmetric ratio of VB-HU at LEV was detected between the mild and severe groups.

**Figure 6:**
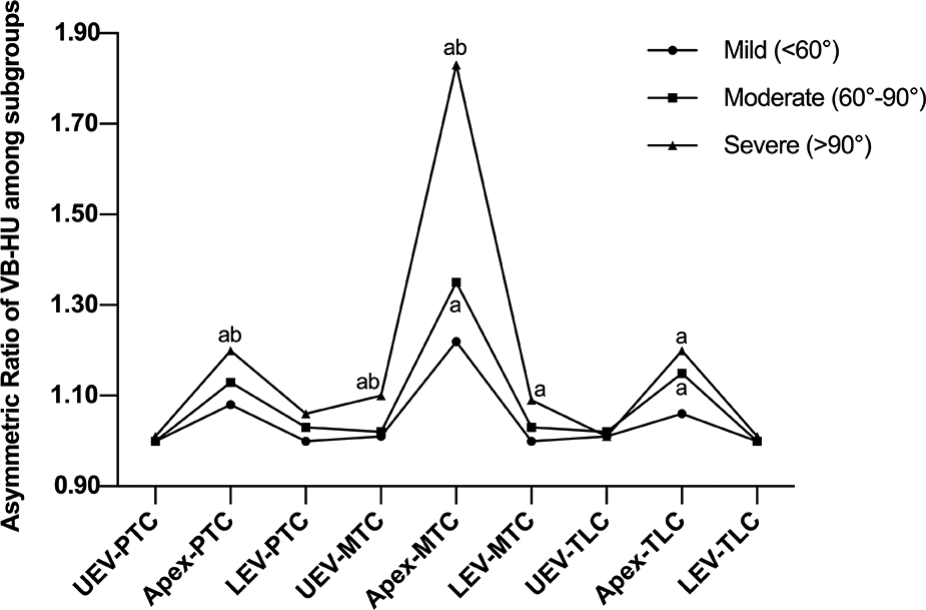
Comparison of the asymmetric ratio of VB-HU among subgroups; a indicates a significant difference compared with the mild group; b indicates a significant difference compared with the moderate group.

For minor curves, the asymmetric HU ratio at the apex of PTC was significantly greater in the severe group, and no significant difference was detected between the mild and moderate groups. However, for TLC, there was no significant difference in the asymmetric HU ratio at the apex between the moderate and severe groups, and the ratio in the mild group was significantly less than that in the other groups. The outcome of VB-HU is shown in Figure 6, and the outcome of PST-HU was similar to that of VB-HU.

## Discussion

The most common tool used for the assessment of BMD is DXA, but the accuracy of DXA-based BMD evaluation can be affected by various factors, such as bony spurs, facet hypertrophy, and vertebral rotation (14). The most accurate site to assess BMD is the cancellous bone, but DXA is not adequate to differentiate between cancellous and cortical bone (18). Thus, DXA may not be reliable for the evaluation of areal BMD in patients with spinal deformities, especially scoliosis (10). The HU value measured by CT scan has been widely proposed for the evaluation of global or areal BMD because it is closely correlated with standard BMD and bone strength (12). With the assistance of multiplanar reconstruction software, it is even feasible to accurately assess the vBMD in patients with severe scoliosis. However, few studies have reported its use in patients with various severities of AIS.

Numerous studies have reported the high prevalence of low bone mass in AIS and the association between BMD and progression of Cobb angle (2, 19, 20). In the current study, low bone mass was detected in 43.0% of patients, and the T-score was significantly correlated with the Cobb angle within the major curve (*r* = −0.633; *P* < 0.01), which was consistent with previous studies. Additionally, this study provided more accurate BMD data of each vertebral body based on HU measurement and suggested that the vBMD at the periapical levels was associated with the curve severity.

The asymmetric vertebral morphology in AIS, including vertebral bodies and pedicles, has been widely reported (21–25). However, the asymmetrical loss of vBMD was only researched in patients with adult degenerative scoliosis (14). Considering the discrepancy in pathogenesis between degenerative and idiopathic scoliosis, this was the first study to demonstrate the asymmetrical loss of vBMD in AIS based on HU measurements. The current study suggested greater VB-HU and PST-HU within the concave side and relatively low HU values within the convex side of the major curve. The asymmetric ratio was the most predominantly presented at the apex and indicated a declining tendency cranially and caudally. Wolff's law may explain this change, as increasing or decreasing the load of bone could remodel the trabecular bone to adapt to these loads (26). In patients with AIS, the gravity load transmitted through the concave side of the vertebra increased and decreased on the convex side, and the apical vertebra of the major curve resisted the most asymmetric loading (27). Such developments occurred not only in the structural curve but also in the nonstructural curve (21). Thus, we measured the asymmetric ratio in all primary and compensatory curves, and similar findings were obtained. The maximum asymmetric ratio was detected at the apex of the major curve, and the degree of intravertebral difference diminished farther away from the apex, presenting symmetric HU values at USV and LSV. However, the asymmetric ratio gradually increased again from the end vertebrae to the apex of the minor curves and decreased from the apex to the end vertebra. The HU values within the concave and convex sides were statistically equal at the UEV of PTC, LEV of TLC, USV, and LSV, which might be attributed to the lower vertebral rotation and asymmetric loading (25).

As the asymmetrical change in vertebra was correlated with the curve magnitude, we included patients who covered the complete spectrum of curve severities from mild (<60°) to severe (>90°). In the current study, the asymmetric ratio of VB-HU and PST-HU at the apex, UEV, and LEV of the major curve and at the apex of the minor curves were significantly associated with the Cobb angle of the major curve, which was in accordance with previous studies (22–25). Similar results were obtained in our subgroup analysis; both the asymmetric ratio and mean VB-HU at the apex of the major curve were significantly different among subgroups. However, when we compared the unilateral VB-HU among different curve severities, no significant difference was detected within the concave side (290.0 ± 31.0 vs. 283.4 ± 30.8 vs. 272.0 ± 51.2, *P* > 0.05), while the VB-HU on the convex side significantly decreased from the mild to severe group (238.9 ± 32.4 vs. 212.3 ± 30.7 vs. 149.9 ± 43.2, *P* < 0.01), which presented as the increasing asymmetric HU ratio mentioned above. These findings suggested that although the loss of vBMD progressed with the curve magnitude, the degree of this change was asymmetrical between the concave and convex sides. The loss of vBMD within the convex side was pronounced, while it was not detectable within the concave side. Additionally, following the progression of curve severity, the aggravation of vBMD loss would be accelerated within the convex side. One explanation for these changes was that more severe scoliosis could cause more gravity shifts toward the concave side, resulting in more asymmetrical compression and shearing force across vertebrae, especially at the apex. UEV and LEV were less translated vertebrae in the major curve, and the results at UEV and LEV confirmed this view, as the difference in HU values was not significant between the mild and severe groups.

Pedicle screws have been widely used in the correction and posterior fusion of AIS because of the adequate three-column fixation provided by insertional torque (28). However, reports have shown that screw loosening is a well-known complication, with a rate of 27%–32% in patients with AIS (7, 15, 29). Since the cancellous bone density of the VB and PST has been identified as a powerful predictor of implant loosening, the asymmetrical loss of vBMD in AIS patients demonstrated by the current study may indicate a high risk of metal implant instability on the convex side around the periapical levels (15). We considered that if surgeons ignored the asymmetric vBMD due to using DXA, the efficacy of fixation could be impacted. Therefore, HU values based on CT scans were recommended as a complement to the DXA T-score for areal BMD evaluation before surgery.

In recent years, the application of CT values to evaluate vertebral bone mineral density has become a research hotspot, especially in lumbar vertebrae, cervical vertebrae, ankylosing spondylitis, and degenerative lumbar scoliosis (30, 31). Our study was the first to use the Hounsfield unit for assessing asymmetrical loss of vertebral bone mineral density and its correlation with curve severity in adolescent idiopathic scoliosis.

## Strengths and limitations

Based on our findings, several strategies should be noted by surgeons. If significant asymmetric vBMD was detected in patients with AIS, the distraction of the pedicle screws at the concave side should be a priority in correcting the major curve, instead of compression of the screws at the convex side (14). Additionally, supplemental anchors (cross trajectory technique) and larger screws should be placed within the convex side around the apex of the major curve to compensate for the impact of low bone mass (32). Keeping an eye on asymmetric vBMD would help surgeons in surgical planning and reduce the risk of screw loosening after surgery. For patients with mild scoliosis, timely exercises, bracing, and appropriate anti-osteoporosis therapy should be performed to eliminate the asymmetrical loss of vBMD, which could delay the curve progression to the surgical threshold (33, 34).

This study has several limitations. First, we recommended placing supplemental anchors and larger screws within the convex side around the apex to provide more fixation, but this was only a hypothesis based on HU measurements and needs to be proven by further biomechanical tests and clinical research. Second, we only evaluated the cancellous bone of PST. In practice, the screw threads could touch the cortical bone when the pedicle width was narrow. Thus, the PST-HU measured in the current study may not be accurate for pedicles with a narrow diameter. Third, only Lenke1–4 AIS patients were included, and whether the results were appropriate for patients with lumbar major curves needs to be confirmed by further research.

## Conclusion

The asymmetrical loss of vBMD was associated with the progression of curve severity in AIS, presenting a slight loss within the concavity and severe loss within the convexity both in major and minor curves. HU values based on CT scans were recommended as a complement to the DXA T-score for areal BMD evaluation before surgery. For patients with severe AIS, the distraction of the pedicle screws at the concave side should be a priority in correcting the major curve, and supplemental anchors, as well as larger-sized screws, should be placed within the convex side around the apex of the major curve to reduce the risk of screw loosening after surgery.

## Data Availability

The raw data supporting the conclusions of this article will be made available by the authors, without undue reservation.
